# 2087 Tool to assess opportunities to augment health literacy and culturally responsive components of research design to enhance diverse engagement

**DOI:** 10.1017/cts.2018.267

**Published:** 2018-11-21

**Authors:** Grisel Robles-Schrader, Ashley Sipocz, Evelyn Cordero, Gina Curry

**Affiliations:** 1 Northwestern University; 2 Office of Human Resources, Northwestern University; 3 Clinical & Translational Sciences Institute, Northwestern University

## Abstract

OBJECTIVES/SPECIFIC AIMS: The goals of this project are to: (1) Help research teams better understand, anticipate, and adapt research to address the needs of diverse communities. (2) Help clinicians and researchers develop patient-centered communication skills needed for more frequent and meaningful engagement of research participants. (3) Identify additional service support needs of clinical research teams not currently offered by other centers (e.g., translation services by certified translators, access to bilingual/bicultural research staff) so they can effectively recruit diverse communities. METHODS/STUDY POPULATION: Mixed methods evaluation approaches centered on obtaining community and academic input aimed at revising the tool to enhance its feasibility and relevance. Round one of focus groups were conducted (4), 2 with a diverse group of community stakeholders, 2 with a diverse group of academic stakeholders. Focus group feedback guided HLCR Assessment Tool revisions. This round of focus groups, served as an opportunity for community and academic stakeholders to discuss shared and divergent priorities related to the development and utilization of the tool. Feedback from these sessions guided a second set of revisions to the tool. Brief surveys were administered at each time point to gather participant demographic data. For the first round of focus groups with community stakeholders, 2 diverse groups totaling 19 people participated (11 female, 7 male, 1 no answer; 6 Asian/Pacific Islander, 6 Black/African American, 4 Latino/Hispanic, and 3 White/Caucasian). Participants served a variety of populations including seniors, youth, underserved, Muslim Americans, Bangladeshi, Arab, South Asian, refugees, community health centers, service organizations, 1st generation students, Latinos, multi-ethnic groups, limited English speaking, people with lupus, un/underinsured, people with HIV, Korean Americans, African Americans, and the disability community. Data pending on the first round of focus groups with academic stakeholders. All participants of the first round of focus groups will be invited to return to a second round of focus groups (2), this time only 2 groups will be held, and these will combine community and academic participants in each focus group. RESULTS/ANTICIPATED RESULTS: Along with formatting and grammatical revisions, recurring recommendations focused on considerations/clarifications in 3 main areas: compensation for all stakeholders, developing a common language and clarifying terms, and aligning the research process with the community. Considerations around compensation was mentioned in discussions related to multiple tool domains. In particular, community stakeholders recommended inclusion and consideration of compensation not just for research participants but also community partners, sites, community representatives, and other academic partners. It was also very important to make sure the form of compensation for both community partners and participants aligns with what was being asked of them. Community stakeholders sited a few examples where they were involved in studies where the time and requirements for participation were not commensurate with the compensation they received or the study budget did not include compensation for community partner effort. Along with edits to questions in the HLCR Assessment Tool, community stakeholders also recommended education for budget/finance personnel on fair compensation for research participants and community partners. In both focus groups, there was also confusion around specific terms and an identified need to develop a common language and clarify terms among all those involved in the research process. More specifically, terms such as community, culture, community of focus, community partners, accessible, and convenient were identified as needing further definition or clarification. Through the focus groups, we learned the valuable lesson that it cannot be assumed broad terms or even seemingly specific ones will be interpreted the same by everyone or have the same meaning in different contexts. Therefore, it needs to be very clear what these terms mean and who or what they represent. Finally, the community stakeholders emphasized throughout both focus groups the importance of making sure that the HLCR Assessment Tool unpack and explicitly emphasize how the research process can align and should align with community needs, communication structures, influencers, and assets. Some factors community stakeholders suggested be considered were: (1) Where the researcher is in the research process; (2) How community members prefer to communicate with each other; (3) Stigma/biases (e.g., class) that may be pervasive in a particular community; (4) Identification of key community influencers/gatekeepers; (5) Learning about a community’s assets along with their needs. DISCUSSION/SIGNIFICANCE OF IMPACT: Currently, there is dearth of resources focused on increasing diverse engagement in clinical and translational research, and consequently, research teams have little or no knowledge or support for how or when to engage community partners in clinical or translational research. The goal of this project is to help fill that gap with a tool to guide clinical and translational research teams in assessing the health literacy and culturally responsive components of their research projects to improve recruitment of diverse populations. Feedback on the first iteration of the HLCR Assessment Tool helped us identify the priorities for community stakeholders and better understand their concerns and needs around engagement with academic partners in clinical and translational research. This understanding will help us enhance the relevance and usefulness of the HLCR Assessment Tool so that clinical and translational science researchers more effectively engage with community partners and help ensure the community’s needs are better aligned with. Therefore, developing and pilot testing this tool can offer a significant opportunity for clinical and translational sciences institutions to enable their researchers and their teams to teams better understand, anticipate, and adapt to the cultural and health literacy needs of diverse populations. More specifically, this tool can: (1) Help clinicians develop the patient-centered communication skills needed to facilitate more frequent and meaningful engagement of potential research participants during medical visits to truly make every healthcare encounter an opportunity for research. (2) Help clinical and translational sciences institutes identify additional service support clinical research teams will need access to in order to effectively recruit diverse communities, that are not currently not supported [e.g., translation services by certified translators, access to bilingual/bicultural research staff at all level (i.e., study coordinators, research assistants, etc.), etc.].

